# Exploring Employee Perspectives on Workplace Technology: Usage, Roles, and Implications for Satisfaction and Performance

**DOI:** 10.3390/bs15010045

**Published:** 2025-01-04

**Authors:** Andreea Barbu, Mirona Ana Maria Ichimov, Iustina Cristina Costea-Marcu, Gheorghe Militaru, Dana Corina Deselnicu, Georgiana Moiceanu

**Affiliations:** 1 Faculty of Entrepreneurship, Business Engineering and Management, National University of Science and Technology POLITEHNICA Bucharest, 060042 Bucharest, Romania; andreea.barbu2901@upb.ro (A.B.); iustina.costea@upb.ro (I.C.C.-M.); gheorghe.militaru@upb.ro (G.M.); dana.deselnicu@upb.ro (D.C.D.); georgiana.moiceanu@upb.ro (G.M.); 2 Academy of Romanian Scientists, RO-050044 Ilfov 3, 050044 Bucharest, Romania

**Keywords:** employee satisfaction, digital solutions, organizational performance, workplace technology

## Abstract

In a dynamic context, where market requirements and needs change often, it is important for companies to adapt to new demands as quickly as possible to continue to be successful. To be able to face numerous challenges, however, organizations need to focus on the needs of their employees, with their satisfaction being an intermediate objective in achieving performance. Since technology is a pillar of each business field, the aim of this study is to explore employees’ perspectives on the use of technology at work, analyzing its main roles within the company, the benefits it provides from the employees’ viewpoint and its implications for satisfaction and performance. To achieve this objective, the authors used existing results in the literature that indicated several methods for the analysis of this subject, and continued with qualitative focus group research that involved six employees working both in hybrid and remote setups, within companies providing services in the engineering area. Thus, in this qualitative study, the needs and preferences of engineering employees are analyzed towards the efficiency of work activities, the improvement of team collaboration, as well as the role of digital technologies in facilitating communication and collecting feedback. Also, the research results address the support of personal and professional development and emphasize the importance of balance between personal and professional life through workplace flexibility—an aspect relatively unexplored in the specialized literature. Apart from the theoretical contributions that the study offers for the development of the literature, the results of this research offer a practical perspective for companies in the field of engineering, suggesting future research directions and recommendations for optimizing performance and increasing the level of employee satisfaction through the implementation of appropriate and personalized digital solutions.

## 1. Introduction

Recently, concerns related to digitization and the use of digital technologies both within the public sector (Haug et al., 2024; Lei et al., 2024) and the private sector (Hammerschmid et al., 2024) have become more severe. The adoption of digital technologies by organizations is not only carried out to pursue the optimization of operational performance (Nusantara et al., 2024), or to increase the security of certain processes within the company or of sensitive data (Danilyan & Dzeban, 2024), but also to comply with laws referring to the protection of personal data (Priyanjani et al., 2024). All the efforts of companies and governments to support the use of digital technologies not only aim to improve organizational performance, but also to increase operational efficiency (Bueno et al., 2024).

In a constantly changing competitive environment, ensuring employee satisfaction is no longer just a choice, but becomes a necessity for organizations. As employee satisfaction refers to how happy and fulfilled employees are in their work environment (Gregory, 2011), prioritizing this aspect becomes one of the core activities of a company, as this satisfaction impacts employee morale (Soetjipto et al., 2021), employee productivity (Najmudin et al., 2024), employee retention rates within firms (Syarif et al., 2024), and overall business success (Azhari et al., 2024).

The Technology Acceptance Model (TAM) is a theoretical framework that explains and predicts how users come to accept and use a particular technology (Davis, 1989). The TAM is based on the premise that two key factors influence the adoption and use of technology: perceived usefulness—the degree to which a person believes that using a technology will enhance their performance or productivity; and perceived ease of use—the extent to which a person believes that using the technology will be free of effort. TAM posits that these perceptions directly influence a user’s attitude toward using the technology, which in turn affects their behavioral intention to use it. Ultimately, this intention predicts actual system use. Over time, TAM has been extended to account for additional variables and contextual factors:

TAM2 (Venkatesh & Davis, 2000), TAM3 (Venkatesh & Bala, 2008), and the Unified Theory of Acceptance and Use of Technology (UTAUT) (Venkatesh et al., 2003), include the following factors: job relevance, cognitive instrumental processes, computer self-efficacy, performance expectancy, effort expectancy, computer anxiety, computer playfulness, social influence, image (social status gained by using the technology), perceptions of external control, output quality, objective usability result demonstrability, and facilitating conditions. The successful adoption of technology, influenced by utility and perceived ease of use, may contribute to the increase in satisfaction at work, since employees are more competent and supported in their daily activities (Giovanni Mariani et al., 2013).

Technologies that offer flexibility and are perceived as easy to use can influence positively the intention to adopt them, allowing employees to manage more efficient tasks and work schedule (Venkatesh et al., 2003).

Building on these theoretical frameworks, this paper seeks to contribute to the field of engineering by exploring employees’ perspectives on the adoption and utilization of digital solutions within the sector. Through a qualitative investigation, the study examines the needs, preferences, and views of engineering employees concerning the implementation and use of digital technologies, as well as their effects on various aspects of work, peer communication, job satisfaction, and work–life balance.

### 1.1. Technology Types and Uses

Technological progress and employee performance are closely correlated (Alam & Murad, 2020; Song et al., 2019). Digital technologies are able to positively influence organizational performance if they are used productively and ethically (Singh et al., 2020). Many companies are starting to invest more and more in digital tools (Verhoef et al., 2021), whether we refer to means of electronic communication (such as e-mail and instant messaging), solutions for online conferences (such as video conferences and chat), platforms for remote collaboration (such as file sharing and shared calendars), and social networks (such as Facebook or Yammer) (Oldham & Da Silva, 2015). These digital solutions can play an important role in cooperation between companies by gathering, storing, and analyzing information (Cherbib et al., 2021), facilitating team interactions, co-creation and knowledge transfer between partners, but also stimulating innovation (Ferreira et al., 2019; Setia et al., 2013).

The use of digital solutions within organizations includes research and development activities, but also project management. Many of the innovative solutions used within companies focus on project design and communication, storage and shared access to files, analysis and data simulations (Marion & Fixson, 2021). In this context, Marion and Fixson (2021) argue that within organizations that focus on innovation processes, four important categories of digital solutions can be identified: technological solutions used for communication (Microsoft Outlook, Google Gmail, Slack, Yammer, Zoom), technological solutions used for product design (Dassault Systems SolidWorks, OnShape, PTC Creo, ANSYS, COSMOS), technological solutions used in project management (Microsoft Project, Teamwork.com, Basecamp), and technological solutions used for data and knowledge management about products (PTC Thing Worx, Dassault Systems SolidWorks PLM, GitHub, Grab CAD).

Vargo et al. (2021) analyze 281 scientific articles from the literature published after 2019 to highlight how digital technologies have impacted people’s lives after the COVID-19 pandemic. Their results identified four major themes: healthcare (143 articles), Education (44 articles), Work (38 articles), Daily life (35 articles). Regarding the types of technologies used after 2019, the authors discovered 15 types of hardware technologies (including wearable devices, webcam-enabled computers, mobile devices, robots) and over 50 software solutions (such as video-based communication platform, tele-work technology, social media, computer or mobile applications, information and datasets). Regardless of the direction followed, the following digital solutions most used for communication were identified: Zoom, FaceTime, WhatsApp, Facebook Messenger, email. Related to telework technology, the following relevant solutions were identified: SPSS, dataset technology, email, online survey, Google sheets. Moreover, Google Trends is used by employees to identify different trends of interest. When it comes to the most popular activities in which technological solutions were used, starting in 2019, Vargo et al. (2021) mention communication first, regardless of the method followed. After communication, for medical services, the technologies used in the provision of medical services were important; for education—the technologies used for teaching and learning; for telework—the use of digital information, as well as the exchange of virtual services; while for daily use—analysis of data and prognosis of COVID-19 cases. The complex analysis of these authors highlights the fact that most of the identified articles focus on the medical field (Senbekov et al., 2020) or the education field (Sun et al., 2020), without many studies that provide a more comprehensive picture of how digital technology was used after the pandemic in all sectors.

Thus, although there are several studies that generally address the types of technologies or their use, there is no detailed research that focuses on the specific way in which employees in the field of engineering, for example, use various digital solutions at work. In addition, there was no analysis of perceptions related to the main aspects of digital solutions that can be used by employees to improve their activities at the workplace.

### 1.2. Employees’ Preferences

As employee satisfaction is an important pillar for improving the performance of a company, it is important to maintain a healthy and productive work environment, but also a thorough understanding of the possible causes that can lead to dissatisfaction among employees. Thus, one of the basic challenges of companies refers to understanding the needs and desires of employees to create a work environment adapted to the period after the COVID-19 pandemic (Weritz et al., 2022). For example, for employees from the younger generations, flexibility is very important (Deal et al., 2010) along with the existence of opportunities for the development of their digital skills (Kane et al., 2017). During this period, employees want more than just to spend their lives working until retirement, so organizations must create a work environment that provides them with perspectives and opportunities for the future (Gratton, 2022).

For companies, it is important to know what the needs and preferences of employees are, especially when it comes to the use of digital solutions, since a mismatch between these needs and the organizational culture applied by the company can cause problems such as resistance to change, communication difficulties and decreased satisfaction in work (Latkovikj et al., 2023). But if these preferences are known, companies could apply more suitable strategies to be able to have more effective collaboration between employees and other interested parties, to increase the level of innovation, but also to increase the level of employees’ commitment.

Recognizing the interaction between IT professionals’ technology preferences and organizational culture is essential for organizations that want to create a harmonious and productive work environment. A misalignment between these factors can lead to a few challenges, including resistance to change, communication barriers, and decreased job satisfaction. On the other hand, strong alignment can lead to improved teamwork, improved innovation, and increased employee engagement (Martins & Terblanche, 2003).

Thus, despite these existing studies in the literature, where the focus is on technological efficiency, there are few studies that present the preferences of employees related to the use of digital solutions at work, especially in the field of engineering. Although companies adopt various types of digital solutions, there are not enough studies to present the perspective of employees related to the existence of certain specifications that digital solutions should have for employees to have a higher level of satisfaction or productivity at work.

### 1.3. Technology Use and Performance

Heslina and Syahruni (2021) focused their attention on the influence of digital technologies, the skills of employees, and on their performance. Based on the results obtained from the regression analyses, the authors demonstrated the fact that digital technology brings important benefits in terms of improving performance at the workplace, especially if it is supported by the company’s management. They mention the fact that it is important for companies to hire competent people in the Human Resources department, who are responsible for ensuring a working climate that supports employees. If this favorable context is created, then the digital technology used by employees leads to the improvement of organizational performance. Sapta et al. (2021) investigated whether aspects such as organizational culture, technology, job satisfaction or self-motivation have a role in improving performance in the banking field. This study highlighted not only the fact that the use of technology has a positive effect on employee motivation at work, but also that it positively impacts employee performance. Moreover, other studies highlight the fact that the use of digital technologies contributes to improving the performance of employees by increasing their productivity (Alam & Murad, 2020; Song et al., 2019).

Bhargava et al. (2021) emphasize that digital technologies help employees to carry out their work and do not suggest replacing them, as they improve technical aspects, eliminating time-consuming routine activities, increasing productivity, and letting employees focus on their soft skills. They also explain that although technology has an important role in workplace productivity, it cannot fully satisfy the emotional needs of employees. Even if companies implement chatbots, the language used by them is not personal and natural, which makes the communication process unsuitable for human emotions (Hill et al., 2015). Therefore, technology could be used to facilitate the communication process between employees, and not necessarily to replace it with artificial intelligence.

Thus, although there are some studies that emphasize the relationship between the use of technology at work and employee performance, there are few that offer a more complex perspective on how digital solutions can be used by employees at an operational level to improve collaboration and communication, to perform tasks efficiently and improve performance. Greater emphasis on communication and collaboration is still necessary to create a better relationship between performance and the use of technology.

### 1.4. Technology Use and Well-Being/Job Satisfaction

Yusuf (2024) researched the relationship between employee commitment, job satisfaction, and companies attentiveness to the needs and preferences of millennial generation employees. The results of his research highlighted that by using and implementing the right technology, companies can meet the expectations of the millennial generation, which will lead to increased employee engagement and job satisfaction for them. Also, creating an open feedback environment improves communication and builds trust, leading to increased millennial engagement and satisfaction. Walker (2024) focuses on the impact that feedback and regular recognition of employees at work have on improving employee satisfaction and operational performance. The results of the study highlight the importance of personalized and empathetic feedback, as well as the strategic use of technology in human resource management. Moreover, paying special attention to the balance between professional and personal life can significantly contribute to improving the engagement and satisfaction of millennial employees (Yusuf, 2024).

Martin et al. (2022) analyze the impact of the use of digital communication and collaboration technologies on the well-being of employees who work from home. Their main results showed that the daily or weekly use of the four types of digital technologies analyzed (collaborative work platform; process automation tools; instant messaging; web conference) leads to an almost immediate increase in productivity at work. However, although productivity is higher, the large flow of information that employees face when they have to use multiple digital technologies leads to an increase in stress at work, as well as to a reduction in their satisfaction. Over time, if employee satisfaction does not improve, labor productivity may decrease (Reinke & Chamorro-Premuzic, 2014).

Axtell et al. (2002) argue that the use of digital technologies contributes to job satisfaction, regardless of the type of company in which they work. Contrastingly, Findlay et al. (2017) underline the fact that certain groups of individuals within the analyzed pharmaceutical field may perceive the implementation of digital technologies in everyday activities in a negative way, especially if they take over a large part of people’s basic activities. Practically, the authors claim that there is a fear for employees that digital technologies (especially those involving automation) can reduce their chances of vertical or horizontal promotion. Therefore, the implementation of these technologies could be a stress factor in the workplace.

Thus, when it comes to the use of technology and employee satisfaction or improving their well-being, there are few studies that analyze this aspect and only mention topics such as communication, collaboration, feedback, and recognition or personal development. There are currently few detailed studies related to how technological solutions can optimize the process of feedback or performance recognition at the workplace, especially in companies that practice hybrid work systems. Also, even if there is an increased interest in professional development, there are still a few studies that show how the implementation of digital solutions within companies supports the continuous development of employees.

### 1.5. Technology Use and Flexibility

Ray and Pana-Cryan (2021) analyze the reality between work flexibility and work-related well-being in the context of digitalization. Their analysis highlights both the positive and negative aspects of work flexibility when working from home, using digital solutions. This flexibility can help many of the employees to take care of personal problems at home (whether it is about taking care of children or the elderly). This positive aspect can help them improve their commitment to the company, increase their job satisfaction and well-being (Kossek & Kelliher, 2023; Wang, 2021). However, not imposing clear boundaries between personal and professional life can lead to negative consequences for employees and their families, if this flexibility affects their time spent with the family (Almer & Kaplan, 2002), particularly as sometimes they will work too much, for 7 days a week for example (Berg et al., 2018). As for flexibility and performance at work, Chatterjee et al. (2022) argue in their study that digital technologies help employees to be more flexible when working from home or at work, which brings significant improvements in organizational performance. Thus, company management should ensure that they choose the best technological solutions so that they can ensure work flexibility, but also improve job performance levels.

Therefore, the Human Resources department should collaborate closely with the company’s management, with the IT departments, but also with the rest of the employees, to be able to pay attention to what digital solutions they propose or choose to implement, as they can change both the well-being of employees, their satisfaction, as well as productivity within the firm (Parry & Battista, 2023). Moreover, the Human Resources department must ensure that the employees are sufficiently prepared to use the digital solutions within the company, so that they can enjoy the benefits brought by technology to the maximum and minimize the associated potential risks with their use.

Thus, although the use of technologies at the workplace and flexibility in the context of teleworking have become topics of great interest recently, there are still a few studies that analyze the relationship between the two concepts, most of them pointing out the advantages or disadvantages of teleworking carried out at home.

Moreover, even if there is a variety of technologies and applications available on the market, their adoption can vary significantly depending on the objectives that the organization sets, as well as the needs and preferences of employees. However, there is still uncertainty regarding the extent to which digital technologies meet the real needs of employees. Furthermore, it is not clear how these technologies can be used to improve communication, collaboration, flexibility in the workplace, feedback and recognition, and personal and professional development.

Therefore, through this study, the authors aim to fill these gaps, providing a more comprehensive and clearer picture of how technology can be used to improve employee satisfaction, but also the overall performance of service companies. Therefore, the authors aim to identify the perspectives of employees within service firms regarding the use of technology in key areas such as communication, collaboration, workplace flexibility, feedback and recognition, and personal and professional development. Moreover, the authors want to analyze how these perspectives align with the applications and technologies most used on the market in these directions.

### 1.6. Rationale of the Study

This study delves into the perspectives of engineering employees on the role of digital solutions in enhancing workplace activities and overall job satisfaction. By focusing on their specific needs and preferences, the research sheds light on how digital technologies can facilitate better communication and collaboration within teams, support feedback mechanisms, and promote both personal and professional growth. Additionally, the paper emphasizes the critical yet underexplored aspect of achieving a balance between personal and professional life through workplace flexibility. The insights gained from this study provide practical guidance for engineering companies to implement effective digital solutions, improve employee performance, and foster satisfaction, while also identifying promising avenues for future research.

In addition, the research is based on specialized literature that explores the adoption of digital technologies, especially through the lens of theoretical models such as the Technology Acceptance Model (TAM) and its extensions (TAM 2, TAM 3, and UTAUT). The present study aims to analyze the proposed subthemes (e.g., communication, collaboration, flexibility, feedback, and employee satisfaction) in relation to TAM principles, thus providing an initial understanding of these aspects from a qualitative perspective.

The results of this focus group not only respond to gaps identified in the specialized literature, but also create a solid foundation for future research. Thus, the authors intend to develop further quantitative research based on the TAM and the results of this study to identify the factors that influence the adoption of digital technologies in companies and how they contribute to improving employee satisfaction and performance. The choice to begin with qualitative research allows for a detailed exploration of the topic, providing a deeper understanding of employee perceptions and clarifying the directions that will be detailed in future research.

The research questions that will guide this study are as follows:

Q1—What are the needs and preferences of engineering employees regarding the use of digital solutions in their daily activities?
Q2—How do digital technology solutions influence employee performance and satisfaction at work?
Q3—What role do workplace flexibility and digital technologies play in improving employees’ work–life balance?
Q4—To what extent do employees perceive digital technology solutions as an effective tool for improving collaboration and communication in their teams?
Q5—How does digital feedback influence employee performance?
Q6—How do digital solutions support the personal and professional development of employees?

Thus, this paper is structured in five parts. In the first section, the introduction and study of the literature is presented, which highlights the gaps in the specialized literature. In the second section, the research methodology is presented, which describes the qualitative research carried out, by describing the most relevant aspects of the conducted focus group. The third section includes the analysis of the results of the qualitative focus group research. The fourth section presents discussions related to the results obtained and the last section emphasizes the conclusions of this study.

## 2. Methods

### 2.1. Focus Group

Considering the theme of this study, the authors decided to use qualitative research based on a focus group, which would highlight the personal and professional experiences of engineering employees regarding the use of digital technologies to improve job satisfaction. The authors chose this focus group technique because it allows for the rapid collection of representative data from a relatively small number of participants, but with experience in a specific field (Richardson & Rabiee, 2001; Rabiee, 2004). Through this method of data collection, participants’ opinions can be analyzed in depth (Babbie, 2020). Another important advantage of focus groups is the fact that they offer participants a higher level of freedom of expression, encouraging them at the same time to interact and debate certain topics brought up by the moderator (Dransfield et al., 2004).

For the organization and development of this focus group, the authors consulted some representative guides and methodologies from the specialty literature (Puchta & Potter, 2004; Breen, 2006; Akyıldız & Ahmed, 2021).

### 2.2. Participants

This research was the focus group type, which aimed to analyze the ways in which digital technology could bring improvements in employee satisfaction. Within this focus group, 6 employees participated, working both in a hybrid and remote regime, within companies providing services in the engineering area.

The number of participants in the focus group was chosen as 6 considering the recommendations in the literature and the specific context of the research carried out. Studies in the literature suggest that focus groups work optimally with between 6 and 8 participants (Gill et al., 2008; Krueger & Casey, 2014; Rabiee, 2004), providing a balance between the diversity of perceptions and the possibility for each participant to contribute significantly (Krueger & Casey, 2014). This size is large enough to capture diverse perspectives, but also small enough to avoid fragmentation of the discussion (Rabiee, 2004).

In addition, the literature supports the use of smaller groups, especially when participants have specialized knowledge or experiences relevant to the subject (Krueger & Casey, 2014; Morgan, 1997). Small groups, between 3 and 6 participants, are recommended in such cases, to facilitate a deeper exploration of the topic and to ensure that each participant can contribute significantly to the discussion (Onwuegbuzie et al., 2009).

Participants need to be familiar with the concepts researched in the qualitative study. This familiarity is essential to ensure the relevance and value of the focus group discussions, as participants with experience in these areas can contribute detailed and well-informed perspectives. Such a selection is supported by the specialized literature, which highlights the importance of selecting participants with specific knowledge to explore the topic in depth (Krueger & Casey, 2014; Morgan, 1997). This approach ensures that the discussions are not limited to general levels of understanding but bring useful information for analyzing the impact of digital solutions in specific professional contexts. In the case of this research, the research topic involves a high familiarity with digital solutions, and the selected participants were chosen for their direct experience in this field, but also for their willingness to participate in qualitative research for scientific purposes. Thus, a group of 6 participants ensure a focused and detailed discussion, without compromising the diversity of opinions. Also, the size of the group was established to efficiently manage the time and resources available, in line with the observations made by Currie and Kelly (2012), as well as Greenbaum (1998), who emphasize the importance of logistical considerations in choosing the number of participants.

In selecting the sample of this research, the following criteria were considered:

- C1—people to be familiar with the concepts of digitization, digital solutions, job satisfaction.
- C2—people to have general knowledge about existing digital solutions within the organizations in which they operate.
- C3—people must have experience in their field of activity of at least 5 years.
- C4—people to have a background in engineering or to work in the field of engineering.
- C5—people willing to participate in this research.
- C6—people should be from Bucharest, be available for 1–2 h to meet with the moderator and the other participants in the “focus group”.

Therefore, the authors employed theoretical sampling in this study, selecting participants with pertinent expertise in the areas of digitalization and digital solutions. 

Theoretical sampling is a method frequently used in qualitative research to explore emerging themes, given that selected participants can bring diverse and informed perspectives to the subject under study (Moser & Korstjens, 2018). This approach helps to obtain deeper information, without necessarily seeking to confirm existing hypotheses, but rather to build a more complete understanding of the phenomenon studied.

Although focus group research has certain limitations, such as high costs, time requirements, and possible biases, the authors applied several measures to minimize them. Participants were selected based on theoretical sampling, given that they possessed relevant expertise in the field of digitalization and digital solutions. Thus, the 6 participants were chosen based on criteria such as at least 5 years of experience in the field, general knowledge of existing digital solutions in their organizations, and specialized training in engineering. The group size was also chosen considering the recommendations of the literature, which suggests that 6–8 participants is optimal to ensure a focused discussion, but also the diversity necessary to capture different perspectives (Krueger & Casey, 2014; Gill et al., 2008; Onwuegbuzie et al., 2009). Although smaller groups can provide a more in-depth exploration of the topic, the authors opted for 6 participants to ensure a balance between diversity and the possibility for each participant to contribute significantly (Rabiee, 2004). Thus, although the research involved certain logistical challenges and limited resources, the authors ensured the validity and robustness of the results through a detailed collaborative analysis of the transcripts, in which all authors reached a consensus on the interpretation of the data.

Thus, in Table 1 some of the most relevant characteristics of the 6 participants can be seen in the focus group.

At the beginning of the study, the moderator explained the main objective of this study is and the main topics that they will address during the meeting to the participants. He also explained that they had to give honest and direct answers about their thoughts, the study itself being based on the analysis of their perceptions, for which there could be no right or wrong answers given (Elghannam et al., 2019).

### 2.3. Focus Group Structure

The focus group thematic discussions were divided into 7 sections (Table 2).

Each section of the focus group had the aim of covering one of the secondary objectives. The conversations were based on the original set of leading questions that were displayed in Table 2. But as the focus group went on, follow-up questions arose organically from the conversations to delve deeper into or elucidate certain points raised by the participants. In addition to capturing subtleties that might not have been initially expected, these emergent questions were intended to improve comprehension of the concepts. The focus group questions are all listed in Appendix A, including the leading questions from Table 2 and the supplementary questions. By addressing the leading questions, the authors responded to these objectives and highlighted their importance for the current research as follows:

The first secondary objective “Determine the presence of digital tools in companies”. is linked to Section 1—Current use of technology. Here, the main question concerns whether employees use digital tools and solutions in their daily work activities. Therefore, participants were asked how the employees in the department/company currently use digital technology in their work. Aspects related to the existence of certain digital tools/solutions that employees use more often were tracked, as well as their opinion related to the most important directions that digital tools/solutions could improve in their work or that of their colleagues. The adoption of digital tools and solutions is highly valuable for companies as it may lead to improved efficiency by automating tasks, allowing key resources to concentrate on other activities. Also, the collaboration between resources and departments is improved as documents and projects can be shared and accessed by everyone who has the rights, also increasing the security level. All these may enhance the overall experience for employees.

The following secondary objective is to “Establish the main needs and preferences of employees in companies”, related to Section 2—Employee needs and preferences. Here, the question raised is the following: which are the current needs that should be covered for an employee to increase that resource’s productivity? Therefore, this section aimed to identify the main needs or preferences that the focus group participants expressed regarding the use of digital solutions at work. In this sense, aspects related to the functionalities that they consider essential when it comes to a digital solution that could help improve their or their colleagues’ productivity and satisfaction at work were tracked. By knowing the needs and preferences, the company makes informed decisions regarding the adoption of new digital tools and solutions. This can have a positive impact on the employees’ performance as they will feel valued, and their opinions listened to. Their results may improve since they will use solutions that are fitted to their needs and level of knowledge.

The next secondary objective is “Establish if digital tools have a positive impact on communication and collaboration inside companies” and associated with Section 3—Communication and collaboration. The main query here is whether the adoption of digital tools and solutions can improve the communication and collaboration of employees. Therefore, the authors wanted to discover if digital solutions are used in the companies where the participants work to achieve better communication or collaboration between colleagues/teams/departments. Also, the authors wanted to see if the participants in the focus group considered these tools to be effective or if there should be other functions that they should fulfill to ensure even better collaboration and communication. The adoption of these tools and solutions by companies, taking into consideration the needs of their employees, may facilitate the fast exchange of valuable information between employees, departments, and other stakeholders. Teams can be coordinated efficiently, and projects managed in real time, from anywhere, facilitating the accomplishment of common objectives and increasing productivity.

The following secondary objective is to “Determine if digital tools can support flexibility in the workplace”, which is linked to Section 4—Flexibility at work. The main question here resides in knowing if these types of tools may lead to better flexibility for employees. Therefore, we tracked how the digital solutions used by the company can support flexibility in the workplace (such as the possibility to work from home, in a hybrid system, to have flexible working hours), as well as the types of tools/digital solutions that could be used to help employees better manage their schedule and achieve a balance between personal and professional life. The flexibility provided by these tools means access to information at any time, from anywhere, allowing employees to collaborate efficiently with their colleagues without being at the office. This type of flexibility has a beneficial impact on the level of satisfaction and motivation of employees since they are more capable of balancing their personal and professional life, reducing stress, without compromising their quality of work.

The fifth secondary objective is “Establish the use of digital tools in collecting feedback from employees in companies”, associated with Section 5—Feedback and Acknowledgment. In this section, the authors wanted to discover if, in the companies where the participants work, there is a practice of giving or requesting feedback to colleagues/superiors and how this feedback is collected. The possible types of digital solutions/technologies that could be used by the company to collect this feedback more effectively, as well as their role in improving employee motivation, were also tracked. Collecting feedback in a centralized place offers a constant flow of information that can be used to identify and take immediate measures to correct areas where issues are found. Even more, this stimulates engagement, transparency and creates an environment of continuous learning by recognizing the human resources performance and a culture of constant improvement.

Another secondary objective is “Determine the impact of digital tools on personal and professional development of employees in companies”, represented by Section 6—Personal and professional development. In this section, the analysis of how digital technology could contribute to the satisfaction of the most important personal and professional needs of the focus group participants and their colleagues was traced. These tools can help companies by offering access to educational resources and personalized evaluations for employees and allow them to develop new abilities and competences. Digital tools and solutions can monitor progress and create personalized development plans, contributing to increasing the knowledge of employees and their ability to adapt to the ongoing changes in industry.

The last secondary objective is to “Determine the benefits brought by the adoption of digital tools and technologies in companies”. This is found in Section 7—Other observations. Therefore, in this last section, the authors wanted to discover other aspects that the participants in the focus group could have considered important, in terms of identifying the ways in which digital technology could bring improvements in the level of employee satisfaction (apart from current use, needs and preferences, communication and collaboration, workplace flexibility, feedback and recognition, personal and professional development). This may help identify new angles of how these types of tools and solutions may positively contribute to the well-being of an employee. Assuring that the environment is beneficial, the company will have engaged employees, that will bring better results and achievements, reflected in the overall performance of the company and the fulfillment of its short- and long-term goals.

It is important to note that qualitative research does not imply generalization, but a deep understanding of the specific context under study. In this case, the statements apply to employees working in the engineering field or in companies providing services in this field. As for non-field employees, the use of digital technologies may vary depending on the nature of the job, but the study was focused on those employees who are directly involved in activities including digital technology for work efficiency or for remote communication. Thus, similar research that would analyze non-field employees could address these issues from a different perspective, considering the specifics of their activity.

### 2.4. Data Analysis

The focus group was held in Romanian, which is the native language of the participants. All discussions were recorded and transcribed in a form containing all the main questions from the study. Data analysis was performed using the thematic analysis method (Braun & Clarke, 2006), a well-established tool in qualitative research.

Thus, the six specific steps of this method were followed: (1) familiarization with the data—by reading the transcripts and repeatedly listening to the recordings to verify the correctness of the text; (2) generation of initial codes—by identifying relevant elements in the participants’ responses; (3) search for themes—by grouping the codes into broader thematic categories; (4) review of themes—by checking their consistency and clarity; (5) definition and naming of themes—by reaching a consensus between the authors on the interpretation of the data; and (6) writing the final results.

First, these transcripts were distributed to 3 authors for the analysis section. Each of them analyzed the transcripts and grouped the information into common blocks, depending on the types of answers given by the focus group participants, analyzing and interpreting data from one’s perspective, without external influences. After the individual analysis of the transcripts, all the authors came together to compare and discuss the results obtained. After the discussion and analysis of these results, the authors reached a consensus on the interpretation of the data, proceeding to present the results obtained following this analysis. This type of analysis is often used in qualitative research, with it being useful to improve the robustness of the results (Hussein, 2009).

## 3. Results

This section may be divided by subheadings. It should provide a concise and precise description of the experimental results, their interpretation, as well as the experimental conclusions that can be drawn.

### 3.1. Current Use of Technology

First, the authors analyzed the perspective of the employees regarding the use of technology through focus group experience. Thus, the first direction of research focused on the current use of technology. In this regard, focus group participants were asked how they and their colleagues use digital technology in their work.

*“…All employees in the company use digital technology daily, probably due to the specific nature of the activity carried out within the company…”*

*(P3)*

*“…Every day, almost all the work is done using electronic means…”*

*(P6) [as reported by participants, depending on the specific nature of their roles]*

All participants highlighted the fact that digital technologies are used in their professional activity (“Every day, almost all the work is done using electronic means”), regardless of the field or type of work (physical, remote, hybrid). They mentioned that technology is used to ease workload, automate certain activities, or even facilitate faster development of products or services offered by the company (Automation/easing of workload is attempted by any accessible digital method). Also, one of the participants highlighted that digital technologies are mainly used in the remote work system for the communication part, which is essential in any field of activity.

*“…Being a telecommunications technology development company, we use a wide range of software to facilitate the rapid development of the company’s internal products as well as those that are offered for sale…”*

*(P4)*

*“…. Efforts are made to automate/lighten the workload through any accessible digital method…”*

*(P1)*

*“…For international communication and ensuring economic traceability, logic, design, etc…”*

*(P2)*

*“…. remote, VPN….”*

*(P5)*

Digital technologies can thus be used both for domestic and international communication, as well as for ensuring the traceability of the activities carried out. Another participant pointed out that sales processes are carried out lately very quickly and efficiently through digital technologies, many of the processes in the company being carried out with the help of digital technologies specific to the activity.

Also related to the use of technology, participants were asked if there are certain digital tools/solutions that they use more often, what kind of solutions they are and how they are used.

*“…From simple excel to temporary tables in a database. They are constantly used by all team members…”*

*(P1)*

*“…Mail, Git, Office, Microsoft Visual Code. Yes, there are tools used only by me: Figma, Sketch…”*

*(P3)*

*“…We all use electronic means of communication (e.g., Microsoft Office suite, Ms. Teams) and, depending on the specifics of the job, other digital solutions: AWS, Java, BI intelligence tools such as Power BI or Tableau, project management tools such as drain…” (as stated by the respondent about their colleagues in the company).*

*“…The main solutions used by developers are divided into 3 categories: office, development and versioning; Office -> any software that facilitates free discussion (the MSOffice suite being the one used by the company given the attractive price and top-down solution | in short, an application for everyone need, from meetings, e-mail and documents); Development -> IDE (Integrated development environment); complete software solution that helps the programmer to create and debug. I will not go into detail.; Versioning -> GitLab; software solution that allows the indexing of all changes made to a software project…”*

*(P4)*

All participants in the focus group highlighted the fact that Microsoft Office suites are used in all companies and in every role as they are affordable, known to all users, and effective. Excel program was mentioned as one of the most useful and handy software tools used in the company, along with all email programs (Yahoo Mail, Gmail, Outlook, etc.). One of the participants in the focus group highlighted the fact that Microsoft Teams is also widely used in companies, especially since the outbreak of the COVID-19 pandemic. The program helps employees in terms of team communication, but also with other stakeholders. As most of the focus group participants are employed in an IT company or in a position in an IT department, they highlighted the fact that the main solutions used by developers are divided into three categories: office, development, and versioning. If the Microsoft Office package has already been mentioned so far, when it comes to development, we are talking about complete software solutions that help the programmer create and debug (IDE—Integrated development environment). Regarding versioning, participants mentioned software solutions that allow indexing of all changes made to a software project, such as Gitlab. Among the other types of digital solutions mentioned, participants also mentioned: Microsoft Visual Code, Figma, Sketch, ENPS editing program and video editing, AWS, Java, BI intelligence tools such as Power BI or Tableau, and project management tools such as Asana.

The last aspect followed when using technology was related to the most important aspects that digital tools/solutions could improve in the activity of focus group participants or their colleagues.

*“…Workload/ avoiding things that are done manually and can be automated…”*

*(P1)*

*“…Easier communication and tractability of projects…”*

*(P2)*

*“…Improving the time allocated for certain requirements, streamlining the way of working…”*

*(P3)*

*“…Reliability, I think it is important that they are easy to access and available. The costs should be bearable for the company and the purchase of user licenses should be easy…”*

*(P6)*

*“…good servers with large storage capacity, VPN, functional platforms specific to the activity…”*

*(P5)*

Their answers highlighted two important directions:

- Improving productivity and organizational performance, by avoiding things that are carried out manually and can be automated, by improving the time allocated for certain requirements, by streamlining the way of working, but also by lowering the costs recorded by the company in the medium and long term.
- Achieving better communication at team and company level, but also through the possibility to access all documents available electronically, important aspects that would make their work easier and make them feel more relaxed within the team.

### 3.2. Employees’ Needs and Preferences Regarding the Use of Digital Technologies

The next topic addressed in this focus group centered on employees’ needs and preferences regarding the use of digital technologies. In this regard, participants were first asked what the main current needs or preferences related to the use of digital solutions can be identified in their companies.

The participants emphasized that they feel the need to participate in trainings to help them better understand and use more efficiently everything related to digitization or automation, including acquiring basic programming knowledge for understanding these tools.

*“…The need for training from several perspectives to learn new things due to digitization/automation…”*

*(P1)*

*“…Programming skills and knowledge of operating systems; also, using basic tools…”*

*(P3)*

They pointed out that it would be better for the company’s activity to be carried out around a single piece of software of reduced complexity, so that they do not have to learn several programs, nor to waste time converting data from one program to be able to use them in another program, so that there is a simplification of the way of working.

*“…To summarize everything in a single software of reduced complexity…”*

*(P2)*

In addition, they believe that if the company uses too many digital solutions, even if they are very good and perhaps effective for certain tasks, familiarity with basic software solutions makes them return to those less efficient solutions.

*“…Another aspect is the familiarity with certain software solutions, there are cases where there are better solutions, but unfortunately, I return to what I know…”*

*(P4)*

Also, one of the participants pointed out a problem he faces, namely that when he uses several software at the same time to process several types of data he needs, the laptop no longer works properly, nor can be used at full capacity.

*“…The nature of my work is limited to 3 software suites that are well defined with many years in the market. The problem I am facing is that when I use them at the same time at the maximum capacity of the used laptop and I am now forced to ask for another one with greater resources…”*

*(P4)*

To prevent such blockages, the focus group participant explained that at certain periods of time, depending on the complexity of the activity, he must request from the company the purchase of a new, more performant type of laptop that can meet existing needs. Also related to this discussion, the other participants added that companies should provide them with digital solutions with high access speed, basic functional platforms with restrictions for certain users to standardize the stored data, but also communication and transparency so they can feel comfortable working with them.

*“…access speed, basic functional platforms with restrictions for certain users in view of the standardization of stored data…”*

*(P5)*

Asked if there are certain functionalities that they consider essential when it comes to a digital solution that could help improve productivity and personal satisfaction at work, focus group participants emphasized interaction with the application. Thus, the ease of use, the appearance of the interface, the simplicity of the work program and the stability of the software are key aspects that must be considered by the company, so that the employees can use the application as easily as possible, not to encounter errors in its software or not to commit errors due to its inappropriate use.

*“…for me, the aesthetics of the application and the user experience are also very important…”*

*(P3)*

*“…Availability and ease of use…”*

*(P6)*

*“…the reaction speed of an action in a tool or file…”*

*(P5)*

*“…The software solution must have the necessary maturity due to the development so that at crucial moments an error does not appear that can cancel hours of work. In personal projects, I will try different new, unique solutions, but when time is critical, I will not base myself on an untested solution…”*

*(P4)*

In addition, one of the participants mentioned the fact that artificial intelligence should be integrated into the digital solutions within the companies, offering translation functionalities assisted by artificial intelligence, to achieve more efficient international communication.

*“…International communication with AI assisted translation…”*

*(P2)*

### 3.3. Communication and Collaboration

The next topic approached in the focus group was communication and collaboration. In this regard, participants were asked if digital solutions are used within the companies in which they operate to achieve better communication or collaboration between colleagues/teams/departments, as well as what these types of solutions are.

All participants emphasized that digital solutions are also used to achieve better communication and team collaboration. In this regard, they mentioned digital tools such as Microsoft Teams, Microsoft Outlook, Google Meet or Zoom, both for communicating with other team members and for communicating with customers. They also mentioned programs such as Agile, Service Now or Jira for developing live plans that can be modified by any department that has access or even the digital HR platform or other personalized communication platforms.

*“…Agile/Service Now/Jira for developing live plans that can be modified by any department that has access…”*

*(P1)*

*“…Microsoft Teams …”*

*(P2)*

*“…we use GIT to version software and create tickets (requirements). For communication we use Google Meet/Zoom/Teams depending on the client…”*

*(P3)*

*“…The office suite offered by Microsoft is sufficient and surprisingly stable in the last year. I don’t think that more is needed in my field…”*

*(P4)*

*“…improvement of applications…”*

*(P5)*

*“…Yes, digital tools are used for Microsoft Teams and Ms. Outlook…”*

*(P6)*

Also related to this aspect, there was the issue of current digital tools and their efficiency, as well as the need for additional functionalities coming from these tools.

*“…they are sufficient together with prior communication for the preparation of plans/tasks per department…”*

*(P1)*

*“…At the moment I think there are enough tools…”*

*(P3)*

*“…Yes, they are effective…”*

*(P6)*

The participants in the focus group emphasized that the digital tools existing within the companies successfully manage to help them in their daily activities, each fulfilling their well-established role. However, one of the participants mentioned that, although there are many popular tools used by companies to organize the team (such as Kanban tool), there are some employees who still prefer to stick to the old techniques of organizing and reporting team activities. This alignment with the latest trends in applications that do much the same thing being considered more a waste of time and a distraction from the core activity of the company.

*“…Now, I cannot provide any other details, since the solutions that are currently considered to bring higher productivity led to the situation of devoting more time to discussion than to implementation. There are a lot of software that teach the last bit of organizing the team using modern techniques (kanban, agile, etc..) and that facilitate the automation of the requirements of these techniques. But for my way of being and those with whom I have worked so far, I have noticed that these procedures do more harm than good…”*

*(P4)*

Regarding the need for new functionalities, only one participant mentioned that perhaps it would be useful to integrate translations made by artificial intelligence into the core activities carried out within the company.

*“…AI translation…”*

*(P2)*

### 3.4. Flexibility in the Workplace

After considering communication and collaboration, we discussed flexibility at work. In this regard, participants were asked if they consider that the digital solutions used by the company can support flexibility in the workplace (such as the possibility to work from home, in a hybrid system, to have flexible working hours).

In addition, they were asked to think about what types of digital tools/solutions they think could be used within the company to help employees better manage their schedule and achieve a work–life balance.

They agreed that digital solutions help them work from home or anywhere else, giving them more flexibility at work. The focus group participants mention that switching to a hybrid work system is one of the best decisions companies could make, emphasizing that now, at least for their specific activity, it is no longer necessary to spend the 5 working days per week from the physical office of the company.

*“…Of course, they can help improve the employee’s program (the hybrid way in my case) …”*

*(P1)*

*“…Yes, the availability of digital communication tools that can also be accessed on the mobile phone allows you some flexibility at work…”*

*(P6)*

*“…Yes, the WFH option is crucial. I can never go back to the office 5 out of 5 days. Productivity increases. The software suite offered by Microsoft (or competitors) is sufficient. After the period of pandemonium that generated this transition, the corporations that own these solutions have invested in the infrastructure and as far as I can see, there are no more periods of downtime. At the end of the day, the most important thing an employee can have is to be motivated professionally and monetarily, but also to have a superior who can understand how to extract his qualities …”*

*(P4)*

The availability of digital communication tools that can also be accessed on the mobile phone allows some flexibility at work, the speed of accessing databases or files stored on company servers is not a problem for these tools. By using these digital solutions, employees have agreed that their schedule is more flexible, and they even have time at home to organize their tasks and breaks differently, so that they can handle small aspects of their personal life, even during the program.

*“…In our field, work flexibility is easy to achieve, and I think the current digital systems help us enough to be able to do a good job. …”*

*(P3)*

*“…the speed of accessing file storage or applications, complete databases…”*

*(P5)*

*“…Assistant AI…”*

*(P2)*

Moreover, some of the focus group participants mentioned that these solutions would give them even greater flexibility if they had integrated aspects related to assistance generated by artificial intelligence (personal virtual assistant).

### 3.5. Feedback and Recognition

The next discussion in the focus group was directed at the feedback and recognition area. Thus, to begin with, participants were asked to think about whether, within the companies where they work, feedback is given or requested from colleagues/superiors.

Among the responses received in this discussion, it is noted that companies request or provide feedback both through 1 on 1 meetings, daily questionnaires sent by e-mail, and by using data collection methods such as the 360 method.

*“…Through 1 to 1 sessions or dailies…”*

*(P1)*

*“…The collection of feedback is done through a standardized process in the industry called 360…”*

*(P4)*

*“…Yes, there is an internal tool for collecting feedback, but it can also be received face to face, directly…”*

*(P6)*

*“…Through different data collection platforms and methodologies…”*

*(P2)*

Also, feedback is very important, this being obtained both during the implementation of the solution and at its end, so that in the next project, employees consider past mistakes and improve their activity.

*“…Feedback is very important, it is obtained both during the implementation of the solution and at the end of it, so that in the next project we can take into account past mistakes and improve…”*

*(P3)*

*“…yes, it is prioritized depending on the case…”*

*(P5)*

When asked about what types of digital solutions/technologies could be used by the company to collect this feedback more effectively, they emphasized that whether it is about collecting online or offline feedback, it is very important that this feedback exists, and, of course, that it is considered. Among the digital solutions they thought of, the focus group participants mentioned the development of special platforms dedicated to collecting feedback, as well as the development and use of analysis matrices with the help of artificial intelligence.

*“…A site that will set goals at the beginning of the year and collect feedback from the supervisor and the people you work with at the end of the year for performance improvement and salary increases…”*

*(P1)*

*“…AI analysis matrices…”*

*(P2)*

*“…I don’t know, we collect the feedback by email…”*

*(P3)*

*“…A more transparent system should be thought of. The problem when you eliminate personal responsibility for a review is that ill-intentioned people can create false-positive reviews. I can’t come up with a better suggestion. I personally believe that the interpersonal relationships of the employees must be formed first and then these solutions are introduced…”*

*(P4)*

*“…recurring questionnaires specific to the targeted team’s activities…”*

*(P5)*

*“…Feedback is encouraged in any form of collection, online or offline, therefore I am not thinking of another digital one…”*

*(P6)*

Participants also pointed out that activities related to collecting and managing feedback could also contribute to motivating employees and increasing their satisfaction at work, especially by providing recognition in case of positive feedback.

*“…Yes, based on them salary increases can be established, personal development activities on the side where necessary…”*

*(P1)*

*“…Always…”*

*(P2)*

*“…Yes…”*

*(P5)*

*“…Yes, feedback in any form helps an employee to develop…”*

*(P6)*

However, one of the participants pointed out that there are some people in executive positions who do not care about this recognition, unlike people in management positions, who sometimes end up working to exhaustion to obtain positive feedback, recognition in front of others and maybe even salary bonuses. Thus, if there is no organizational culture that inspires employees with the same sets of values, it may happen that some do not care about this feedback and frustrations between employees intervene over time.

*“…No, because no one wants to have the responsibility of giving a negative review, but also no one will want to collect this feedback for the purpose of career advancement. When you set a positive feedback target (which will happen if this system is implemented) unnecessary competitiveness will be created. I believe that in the field of development it is necessary to allow evolution; an example is to lose a developer’s salary for a week and implicitly to increase the development time to force him to learn a process or a concept in the field. I worked in a company that was based on this concept of feedback, which at the end is realized in a substate remuneration. The 5 people at the top worked until exhaustion and others (such as me) did not give two cents on the system, which created a major rift between the employees…”*

*(P4)*

### 3.6. Personal and Professional Development

The next topic approached during the meeting was related to the personal and professional development of employees. To begin with, they were asked to think about their most important personal and professional needs at that moment.

In addition to wanting more free time for personal activities, to balance personal and professional life, participants emphasized that their needs focus on two directions: interpersonal relationships and the need to keep up with technology.

*“…Trainings in various useful applications…”*

*(P1)*

*“…Free time…”*

*(P2)*

*“…I think that first of all the need to develop professionally, as well as in human relations…”*

*(P3)*

*“…On the personal development side, it is important to develop continuously, to keep up with new technologies and to deepen your knowledge at work…”*

*(P6)*

*“…meeting deadlines and accuracy in deliverables…”*

*(P5)*

*“…Let’s stay constantly informed and not stop studying. Technology advances faster than an individual’s absorption capacity. The idea is to understand the basic concepts and then apply them to new requirements. As personal needs, it is to succeed in being able to have a balance between work and life so as not to reach intellectual exhaustion…”*

*(P4)*

Thus, for the first aspect, they mentioned the need for training or workshops to help them communicate better and perform better in relationships with colleagues, clients, or other stakeholders. For the second aspect, they mentioned the need for training to understand and use various useful applications and technologies.

*“…The Internet is full of tutorials from how to change a screw to how to describe the architecture of operating systems. These tutorials are necessary to impose themselves in any field. In Romania, we have a multitude of personalities who have reached positions that they can no longer justify. They no longer have the necessary knowledge to perform that job. Constant reevaluation would be a good thing to consider…”*

*(P4)*

When asked how they think digital technology could contribute to meeting these needs, participants answered that companies can provide them with access to online learning platforms such as: LinkedIn Learning, O’Reilly Learning Platform, Udemy, Pluralsight, but they should also consider allocating time for carrying out these activities, as well as allocating budgets in the event of certifications or accreditations that they could obtain at the end of the courses.

*“…These solutions are already implemented in several large corporate companies that offer access to online learning platforms such as: LinkedIn learning, O’Reilly, Udemy, Pluralsight…”*

*(P6)*

*“…Through the theoretical explanation accompanied by practical examples according to it…”*

*(P1)*

They also mentioned that the assistance provided by artificial intelligence could have an immediate beneficial impact, helping them understand certain concepts, procedures or working methods faster.

*“…Assistant AI…”*

*(P2)*

*“…centralized systems, complete databases and new tools…”*

*(P5)*

In the final part of the focus group, participants were invited to add information that they consider important or that had not been addressed so far about how digital technology could bring improvements in employee satisfaction.

From the discussions, one participant emphasized that the Microsoft Office suite should not be missing from any organization. In addition, the other digital solutions that could be adopted by the company to help them must comply with the following criteria:

- Be modern
- Be easy to understand
- Consume relatively few resources
- Use free solutions as much as possible
- Have tutorials created in the last two calendar years.

*“…Today’s technology used in corporations can be summed up in two words: office suite. Satisfaction at work is given by the concept of personal and monetary development. The software solutions that are used must have the following criteria:*

- *They must be modern (if there is no easy-to-find documentation, then it cannot be used)*
- *To be easy to understand (SPSS cannot be used globally due to complexity)*
- *To consume relatively few resources (Not everyone has a high-performance computer)*
- *To use free solutions as much as possible*
- *There must be user tutorials created in the last two calendar years …”*

*(P4)*

*“…At the level of satisfaction, I can observe other requirements: the need for socialization, the need for belonging, the need for performance. I don’t think these needs can be met with the help of current software solutions…”*

*(P4)*

Regarding job satisfaction, participants mentioned that digital technology can also help with wellness/mental health and stress management/work–life balance of employees through access to news platforms with tips and tricks for well-being (e.g., Headspace).

*“…Digital technology also helps with wellness/mental health and stress management/work–life balance of employees through access to news-type platforms with tips & tricks for well-being (e.g., Headspace) …”*

*(P6)*

*“…I believe that software solutions are customized to the needs of the employee and the employer. If you work with data, use Excel, Power Bi. If you work in construction, you use CAD. If you work in games, you use Blender for graphics, etc. But I repeat what I can benefit from the basic requirements of these software numbered above. If any of the respective points are not met, then satisfaction drops drastically…”*

*(P4)*

*“…The idea of feedback is interesting, but I don’t trust the polite way of approaching interpersonal realities; it won’t work. If you burn one, he will burn you. Also, we are not Japanese, we do not have a shogun. If you can steal the top one, you steal it. …”*

*(P4)*

In addition, when it comes to employee satisfaction, it is important to satisfy needs such as socialization, belonging, recognition, which do not necessarily have to be satisfied with software solutions, but it would be much easier to manage both by them and by the company. Thus, if companies want to improve the level of employees’ satisfaction, they should also focus on team building, encouraging teamwork or offering bonuses for performance.

## 4. Discussion

In a dynamic context where digital solutions are organized within companies to bring added value to the company, digitization can be used not only to improve organizational performance, but also to bring positive changes in employee satisfaction.

The results of the focus group research highlighted the significant concern of organizations to implement digital technological solutions that can contribute to the easier development of activities within the company (Urbinati et al., 2020). Focus group participants mentioned different job-specific needs and expectations regarding communication, collaboration, task management, feedback and performance evaluation, personal and professional development, and wellness aspects. In this context, they emphasized that the implementation of digital technology solutions can be perceived as an opportunity to address these needs (Veile et al., 2022), and implicitly to improve the experience (Selimović et al., 2021) and satisfaction of employees within the organization (Bueno et al., 2024).

### 4.1. Practical Implications of Focus Group Results for Companies

The results of this focus group can provide important contributions for companies, directly answering the research questions formulated previously, as follows:

Q1—What are the needs and preferences of engineering employees regarding the use of digital solutions in their daily activities?

The usage of digital tools and solutions in companies will increase operational efficiency by automatization of tasks, reduced time for optimizing flows and administrative processes. Companies become more competitive and may implement rapid change in the market. This is also sustained by the research of Nusantara et al. (2024) and Bueno et al. (2024).

Q2—How do digital technology solutions influence employee performance and satisfaction at work?

Determining the needs and preferences of employees in terms of digital tools and solutions may provide a basis for companies to make informed decisions and adopt personalized ones. This may prevent employees’ resistance to change and improve their retention rate, especially in the field of engineering. Engaged employees have better results and achievements, bringing added value to the company. Walker (2024) also analyzes the importance of constant feedback and how personalized digital solutions may lead to performance and satisfaction of employees as well as their retention rate. Weritz et al. (2022) cover the topic of how personalized digital solutions can sustain productivity and prevent employees’ resistance to change.

Q4—To what extent do employees perceive digital technology solutions as an effective tool for improving collaboration and communication in their teams?

By providing a communicative environment, companies create a culture that supports and encourages collaboration. This type of environment is perceived as friendly by the employee, with access to information in real time, efficient management of distributed teams and improved internal and external communication. Companies may optimize decisional processes and have better response time in projects, delivering the expected results on time. Priyanjani et al. (2024) analyze the way in which digital tools are the basis of distance collaboration and managing distributed teams, highlighting the benefits if real time access to information.

Q3—What role do workplace flexibility and digital technologies play in improving employees’ work–life balance?

The flexibility brought by digital tools and solutions allows the autonomy of an employee and a proper balance between work and personal life. Human resources’ absenteeism is reduced by allowing them to work from a distance, from their own comfort, being more motivated and less stressed, also reducing the operational costs. Gratton (2022) also explores the benefits provided by the flexibility provided through the adoption of digital tools, showing how increased autonomy and reducing absenteeism contribute to motivated employees and lower operational costs for companies.

Q5—How does digital feedback influence employee performance?

Through collecting feedback, companies may interfere to solve potential issues that may disturb the working environment and relations between employees. Since immediate solutions are implemented, the engagement and performance of employees are maintained at a high level by supporting and acknowledging their opinions. Sapta et al. (2021), as well as Heslina and Syahruni (2021), debate how the usage of digital tools to collect feedback influences the performance of employees by providing recognition and engaging their commitment. Also, this feedback identifies and solves potential issues which contribute to the performance of employees.

Q6—How do digital solutions support the personal and professional development of employees?

The personal and professional development of employees is very important for companies as they are key resources in each organization. Allowing employees to grow and supporting them into areas that they want to delve into will lead to competent and prepared teams that can face any challenge. Barbu et al. (2024) analyze the positive impact of technology on the personal and professional development of employees. According to their study, digital tools are contributing to the overall performance of organizations. Danilyan and Dzeban (2024), debate how digital technologies sustain professional development and how companies may assure the development of competence through a safe online environment.

### 4.2. Theoretical Implications of Focus Group Results for Specialized Research Literature

The findings about the use of digital tools and solutions in companies may contribute to the specialized research literature by analyzing the way in which digitalization transforms organizational structures and business models, highlighting how technology can redefine organizational culture and sustain the concept of digital transformation. Research in this direction is also developed by Hammerschmid et al. (2024) and Vial (2021) which provides a theoretical framework to better understand the impact of digitalization on companies, and demonstrates how technology changes organizational culture and business models.

The needs and preferences of employees cover the importance of user-centered design and human–computer interaction in the research field. It offers a framework for understanding the way in which digital tools and solutions impact the individual and team performances of employees. Here, Weritz et al. (2022) also make reference to human–computer interaction as digital tools may increase the overall performance of teams and employees. Walker (2024) highlights the necessity of taking into consideration this type of interaction as a user centered design can facilitate the interaction between employees and technology, having a positive influence on performance.

Digital tools and solutions, from a research point of view, contribute to the academic literature on collaborative technologies by analyzing how technology influences the organizational communication and the structure of teams, highlighting the collaborative dynamics and technological efficiency in distributed teams. Cherbib et al. (2021) and Schwarzmüller et al. (2018) also study the influence of digital technologies on the structure of teams and how they sustain efficient collaboration to better understand the dynamics and importance of technology in modern companies.

Flexibility is a term that is frequently debated in the specialized literature towards concepts like remote work and work–life balance. It underlines the positive impact of flexibility on employee satisfaction and long-term performance. This is also studied by Chatterjee et al. (2022) and Ray and Pana-Cryan (2021), who explore the impact of remote work on organizational performance and the positive effects of flexibility on the well-being of employees, which can be seen in an increased level of satisfaction and performance of them.

Collecting feedback is related to employee engagement and it explores the role of digital tools in evaluating performance, emphasizing the benefits of constant monitoring and its impact on improving employee’s motivation. Syarif et al. (2024) explored this topic through their study, reaching the same conclusion. Yusuf (2024) also develops research in this direction, his findings sustain the importance of collecting feedback through digital tools on the improvement of employees’ abilities and motivation.

In the literature, concepts such as lifelong learning and e-learning underlined the impact of digital tools and their solutions, as well as their role in adapting to ongoing and rapid changes in the business environment. The research of Ferreira et al. (2019) and Setia et al. (2013) also offer a solid theoretical background for digital tools and solutions in sustaining life-long learning and e-learning, key elements for companies to adapt to the on-going business environment.

The theoretical implications of this study are essential for deepening the understanding of the impact of digitalization on organizations, as well as for building a solid foundation for future research. In particular, the results obtained from the focus group analysis will contribute to the development of a clearer and more detailed theoretical framework for future quantitative research, which will aim to determine the factors influencing the level of adoption of digital solutions in engineering organizations, with the aim of increasing employee satisfaction. This quantitative research will be based on the theoretical framework of the Technology Acceptance Model (TAM) and will use the results of the present study as an empirical basis to analyze in more detail the variables involved in the acceptance of digital technologies.

Thus, this study will support a better understanding of the factors determining the adoption of technologies within engineering companies and will address issues such as employee performance, job satisfaction, and the impact of digital technologies on them. The study will contribute to the literature by exploring how the variables involved in TAM (Perception of Usability, Perception of Ease of Use, Intention to Use, etc.) influence organizations’ decisions to adopt digital solutions and how these decisions impact employee satisfaction in engineering organizations.

## 5. Conclusions

The current study offers significant contributions by presenting specific results for the engineering area, which indicate the key procedures and know-how of implementing technology in this field, including the most used types of digital solutions, but also important key aspects should be maintained so that employees’ performance can be improved at the workplace. Through this study, we included the perspective of the employees in the engineering area about their preferences and main needs at work that can be satisfied by the digital solutions implemented by the companies. Even more, the main functionalities of these digital solutions are highlighted, which helps employees to improve their activity at the workplace, giving them a sense of satisfaction at the same time.

As was presented throughout this paper, in the specialized literature there are numerous studies that present different aspects related to improving the performance and satisfaction of employees through digital technologies. However, these studies do not comprehensively integrate the employees’ perspective with the concrete data about the digital solutions used in practice. Thus, the results of this paper contribute to the expansion of knowledge in the field, providing relevant information related to how technologies can support essential areas such as communication, collaboration, workplace flexibility, feedback and recognition, as well as personal and professional development. The analysis of the preferences and needs of the employees, as well as the five themes followed, can be the basis of some practical recommendations for organizations in the engineering field.

The results of the study highlight the fact that technology must be included a company. Even the use of basic digital solutions such as the Microsoft Office suite can help employees within companies to be more organized, more productive, and less stressed due to manual tasks. The results also highlight that the digital solutions used by the company should be modern but easy to understand, with tutorials available if necessary, and consume few resources. By using solutions that can help employees communicate and collaborate more easily, managing tasks and projects better, collecting and providing feedback, helping them with personal and professional development, and taking care of their well-being, companies can benefit on many levels. Although studies have shown that digital technology solutions used by companies can contribute not only to improving employee performance, but also to improving employee satisfaction, they highlight the fact that it still takes longer for digitalization to produce significant effects in terms of employee satisfaction.

The results of this research must be interpreted carefully, however, as the research also has some limitations. First, the employee’s perspective concerned the opinions of a small number of engineering employees. In this respect, their perceptions regarding the experiences they had within the companies in which they operate were interpreted. Even though the six-person sample size may be viewed as a limitation, it is appropriate for focus group research. Smaller groups (e.g., six–eight participants) are recommended by literature as the best way to achieve depth and diversity of opinions without sacrificing the caliber of conversation (Krueger & Casey, 2014). Additionally, smaller focus groups (three–six members) are advised to enable in-depth examination of themes in situations where participants possess specialized knowledge (Krueger & Casey, 2014; Morgan, 1997). For the future, quantitative research that collects the perception of a much larger number of participants, from several fields of activity, would provide significant additions to improving employee satisfaction through digital technology solutions. Secondly, only one perspective was analyzed in this study, therefore, a market perspective was missing. Thus, for the future, it could be necessary to fill the gap related to the correlation of the needs and preferences of employees with the technological solutions available on the market. In this regard, the exploration of employees’ perspectives should be completed using an exploration of market offers (an analysis of the most used technological applications identified on the market).

As for future research directions, the authors can focus on several routes. Firstly, they could carry out empirical studies that include a wider sample of companies from various industries and geographical regions, to validate and generalize the results obtained. Secondly, separate studies can be conducted on emerging technologies (such as artificial intelligence), and how they can be adopted and used to improve employee satisfaction and organizational performance. Thirdly, it would be useful to carry out a comparative analysis regarding the impact of digital solutions used by companies on different generations of employees. Thus, these directions could offer additional relevant perspectives for company management to make the best decisions to maximize the use of digital solutions within companies, with a positive impact on employees as well.

## Figures and Tables

**Table 1:** Focus group participants.

P	Gender	Age	Domain of Activity	Position	Experience in the Field (Years)	Type of Work
P 1	M	25	Cyber security	It Systems Analyst	5	Hybrid
P 2	M	35	Engineering	Lead Engineer	10	Remote (work from home)
P 3	M	31	Custom software development	Administrator	13	Hybrid
Pt 4	M	33	IT-programming	Developer	7	Hybrid
P 5	F	32	IT-programming	Business Analyst	9	Hybrid
P 6	F	31	Engineering and Management	Project Manager	9	Hybrid

Source: Authors’ contribution.

**Table 2:** The leading questions used in the focus group.

Objectives	Sections	Leading Questions
Determine the presence of digital tools in companies	Current use of technology	How do employees in your department/company currently use digital technology in their work?
Establish the main needs and preferences of employees in companies	Employee needs and preferences	At this moment, what are the main needs or preferences that you show regarding the use of digital solutions at work?
Establish if digital tools have a positive impact on communication and collaboration inside companies	Communication and collaboration	At this moment, within your company, are digital solutions used to achieve better communication or collaboration between colleagues/teams/departments?
Determine if digital tools can support flexibility in the workplace	Flexibility in the workplace	Do you think that the digital solutions used by the company can support flexibility in the workplace (such as the possibility to work from home, in a hybrid system, to have flexible working hours)?
Establish the use of digital tools in collecting feedback from employees in companies	Feedback and recognition	Within the company where you work, is the practice of giving or asking for feedback to colleagues/superiors a common one? How is this feedback collected?
Determine the impact of digital tools on personal and professional development of employees in companies	Personal and professional development	Right now, what do you consider to be the most important personal and professional needs of you and your colleagues? How do you think digital technology could help meet these needs?
Determine the benefits brought by the adoption of digital tools and technologies in companies	Other observations	Are there other aspects that you consider important that have not been covered in this discussion in terms of identifying ways in which digital technology could bring about improvements in employee satisfaction?

Source: Authors’ contribution.

## Data Availability

Data are contained within the article.

## Appendix A. The Main Questions Used in the Focus Group

**Sections**	**Leading Questions**
Current use of technology	- How do employees in your department/company currently use digital technology in their work? - Are there certain digital tools/solutions that you use more often? - What do you think are the most important aspects that digital tools/solutions could improve in your work or that of your colleagues?
Employee needs and preferences	- At this moment, what are the main needs or preferences that you show regarding the use of digital solutions at work? - Are there certain features that you consider essential when it comes to a digital solution that could help improve your or your colleagues’ productivity and satisfaction at work?
Communication and collaboration	- At this moment, within your company, are digital solutions used to achieve better communication or collaboration between colleagues/teams/departments? - What other functions do you think digital solutions in the company should fulfill to ensure even better collaboration and communication?
Flexibility in the workplace	- Do you think that the digital solutions used by the company can support flexibility in the workplace (such as the possibility to work from home, in a hybrid system, to have flexible working hours)? - What types of digital tools/solutions do you think could be used within your company to help employees better manage their schedules and achieve work–life balance?
Feedback and recognition	- Within the company where you work, is the practice of giving or asking for feedback to colleagues/superiors a common one? How is this feedback collected? - What types of digital solutions/technologies could the firm use to collect this feedback more effectively? - Do you think these activities could also help motivate employees by providing recognition for positive feedback?
Personal and professional development	- Right now, what do you consider to be the most important personal and professional needs of you and your colleagues? - How do you think digital technology could help meet these needs? What types of digital resources, solutions or technologies do you think could help employees within the firm improve their skills and advance their careers within the firm?
Other observations	- Are there other aspects that you consider important that have not been covered in this discussion in terms of identifying ways in which digital technology could bring about improvements in employee satisfaction?
